# Physical Robustness and Resilience Among Long-Lived Female Siblings: A Comparison With Sporadic Long-Livers

**DOI:** 10.1093/geroni/igaa057.1696

**Published:** 2020-12-16

**Authors:** Angéline Galvin, Svetlana Ukraintseva, Konstantin Arbeev, Mary Feitosa, Kaare Christensen

**Affiliations:** 1 University of Southern Denmark, Odense C, Denmark; 2 Duke University, Durham, North Carolina, United States; 3 Washington University School of Medicine, St Louis, Missouri, United States; 4 University of Southern Denmark, Odense C, Syddanmark, Denmark

## Abstract

Background: Long-lived individuals are central in studies of determinants of healthy longevity. However, few pro-longevity factors have been identified, presumably because of “phenocopies”, i.e. individuals that live long by chance. Familial longevity cases may include less phenocopies than sporadic cases and provide better insights into longevity mechanisms. Here we examined whether long-lived female siblings have a better ability to avoid common diseases at ages 65+ (proxy for “robustness”) and/or survive to extreme ages (proxy for “resilience”) compared to sporadic long-livers. Methods: 1,156 long-lived female siblings were selected from three nationwide Danish studies (DOS, GeHA, LLFS) and age-matched with sporadic long-lived female control from the Danish population. Outcomes included cumulative incidence of common health disorders from age 65, and overall survival from 2006 onwards. Logistic and Cox models were used to evaluate incidence and survival respectively. Results: Long-lived female siblings had significantly lower risks of hypertensive (OR=0.84; 95%CI=0.71-0.99) and cerebrovascular (OR=0.73; 95%CI=0.55-0.96) diseases and depression (OR=0.74; 95%CI=0.62-0.88) at ages 65+, and better survival to extreme ages (HR=0.71; 95%CI= 0.63-0.81) compared to sporadic long-livers. After adjusting for diseases above, the association with mortality changed only marginally (HR=0.73 (0.64-0.83)). Conclusion: Familial longevity cases could be more informative for studying mechanisms of healthy longevity than sporadic cases. Long-lived female siblings demonstrate better physical robustness and resilience than their age-peers from general population, which might be attributed to a genetic component in familial longevity.

